# Editorial: Regulation of Antibiotic Production in Actinomycetes

**DOI:** 10.3389/fmicb.2020.01566

**Published:** 2020-07-22

**Authors:** Yvonne Mast, Evi Stegmann, Yinhua Lu

**Affiliations:** ^1^Department Bioresources for Bioeconomy and Health Research, Leibniz Institute DSMZ -German Collection of Microorganisms and Cell Cultures, Brunswick, Germany; ^2^Department of Microbiology, Technical University Braunschweig, Brunswick, Germany; ^3^German Center for Infection Research (DZIF), Partner Site Tübingen, Tübingen, Germany; ^4^Department of Microbial Bioactive Compounds, Interfaculty Institute of Microbiology and Infection Medicine, Faculty of Science, University of Tübingen, Tübingen, Germany; ^5^College of Life Sciences, Shanghai Normal University, Shanghai, China

**Keywords:** actinomycetes, antibiotic, secondary metabolism, regulation, signaling

Actinomycetes are a well-known resource for the discovery of novel bioactive compounds. Up to two-thirds of all antibiotics in use today are natural products from actinomycetes or semi-synthetic derivatives thereof (Barka et al., 2016). *Streptomyces* species have thus far proven to be a particularly useful resource. It is now 80 years ago (1940) when Selman Waksman isolated the first antibiotic from an actinomycete's species, which was actinomycin A from *Actinomyces* (*Streptomyces*) *antibioticus* (Kresge et al., 2004). Shortly after, he coined the term “antibiotic,” which is just one reason why he is considered widely to be the true “Father of Antibiotics” (Kresge et al., 2004). In the following years Waksman's group successfully applied his screening technique for the identification of natural substances and isolated more than 20 additional new bioactive compounds, thereby initiating the so-called “golden era of antibiotic discovery.” One of the most important Waksman compounds was streptomycin, which Albert Schatz and he identified in 1943 as a product from *Streptomyces griseus*. As discoverer of the “first antibiotic effective against tuberculosis” he was rewarded with the Nobel Prize in 1952 (Kresge et al., 2004). Since that time, actinomycetes, especially streptomycetes, have been studied intensively, not only because of their gifted metabolic potential but also due to their interesting biological features: e.g., streptomycetes have extraordinarily large genomes with up to ~13 Mbp, which are of linear style and with a high GC content (>70%). They are characterized by an unique life-cycle, involving the formation of mats of fungus-like mycelium from which aerial branches are formed that carry chains of spores. The spores are then readily released for dissemination (Chater, 2016). Nowadays, actinomycetes are still, or maybe more than ever, a valuable resource for novel drugs. New antibiotics are urgently needed due to the constant rise of infections caused by drug-resistant pathogens (O'Neill, 2016). In recent years, a wealth of expertise has been gained regarding especially knowledge on antibiotic biosynthesis in these organisms. Particularly since the advent of next-generation sequencing technologies, the field of antibiotic research has experienced a remarkable revival, which is due to the upcoming of a large amount of genome sequence information that gave rise to novel approaches in order to exploit actinomycetes for drug discovery. Comparable few studies focus on a better understanding of regulatory mechanism of antibiotic biosynthesis. Understanding regulatory principles in antibiotic producing organisms is, however, fundamental for targeted optimization of antibiotic production yields, activation of silent gene clusters to find novel antibiotics, or facilitating genetic engineering approaches, e.g., to allow heterologous expression of engineered gene clusters. Besides that, regulation is the interface connecting aspects of chemical differentiation with complex morphological differentiation events in actinomycetes. Thus, understanding regulation means also learning about actinomycetal biology.

Regulation of secondary metabolism in actinomycetes is a complex process depending on various factors, such as nutrient limitation, oxygen supply or pH conditions (Liu et al., 2013; Wohlleben et al., 2017). Regulation can occur at the level of transcription and/or translation. Concerning the former, regulatory signaling cascades can include global, as well as pathway-specific (cluster-situated) transcriptional regulators. Thereby, various chemical substances may serve as effectors for transcriptional regulators, such as quorum-sensing-like γ-butyrolactone (GBL) molecules, antibiotics, or intermediates thereof. Groundbreaking scientific achievement in this field has been made already in the 1980s by Sueharu Horinouchi using the above mentioned famous *S. griseus* strain (Ohnishi, 2010). Horinouchi et al., showed that streptomycin production and morphological differentiation of *S. griseus* is under control of a complex regulatory signaling cascade triggered by the microbial hormone-like substance A-factor (Horinouchi et al., 1983; Horinouchi, 2002). The A-factor is a GBL molecule and was initially discovered in the 1960s by Khokhlov et al., as a substance controlling streptomycin production in the originally named strain *Actinomyces streptomycini* (*S. griseus*) (Khokhlov et al., 1967). Horinouchi thereafter worked out in detail that the A-factor is produced in a growth-dependent manner at nanomolar concentrations. When A-factor concentrations reach a certain threshold the cognate repressor-type receptor ArpA dissociates from its target gene *adpA*. *adpA* itself encodes the transcriptional regulator AdpA, which acts as a pleiotropic regulator controlling several hundred genes in *S. griseus*, including a number of genes involved in morphological differentiation and secondary metabolite biosynthesis (Horinouchi, 2002). The A-factor signaling cascade became a model system for understanding regulatory principles in antibiotic-producing actinomycetes. Indeed, GBLs are produced by many, if not all streptomycetes (Bibb, 2005), whereby the signaling cascades that are governed by GBLs and which control transcriptional activation of antibiotic gene clusters can have different compositions in various antibiotic producers (Bibb, 2005; Wohlleben et al., 2017). Furthermore, not only GBLs may function as signaling molecules but also antibiotics or their intermediates, which altogether result in complex regulatory networks including elaborate feedback and feedforward loops (Xu and Yang, 2019). Besides that, such networks themselves are embedded in larger regulatory circuits involving transcriptional as well as translational regulation, which may respond to diverse environmental signals as mentioned above (Bibb, 2005; Liu et al., 2013). Most of these various regulatory interactions have not been explored yet. Thus, there is still a lot to investigate for actinomycetes.

With this Frontiers Research Topic we aimed to create space for current advances and knowledge on regulation of antibiotic biosynthesis in actinomycetes and highlight the importance of this topic for antibiotic research. We collected 13 articles, including nine original articles and four reviews covering a variety of different regulatory aspects of antibiotic production. The articles address the topics of regulatory networks, signaling molecules for antibiotic production, studies on regulator-driven activation of silent gene clusters, optimization of antibiotic production, as well as regulatory effects from small open reading frames (ORFs) and small non-coding RNAs in actinomycetes.

The overarching review article from Juan Martin and Paloma Liras reports on the interplay between various environmental stress signals (e.g., variations in carbon and nitrogen sources, phosphate, oxygen, iron, and other nutrients) and major transcriptional factors in actinomycetes. It is described how these different signals are integrated at the molecular level (Martín and Liras). The authors propose a cross-talk of the transcriptional regulators, which bind to specific DNA regions, so-called “integrator sites,” that allow to compensate for unbalances produced by metabolic stresses—a phenomenon designated as the “balance metabolism safety net” (Martín and Liras). The review article from Kong et al., concentrates on autoregulator-driven regulation of secondary metabolite biosynthesis. Here, the authors highlight the recent findings on regulation of antibiotic biosynthesis by small molecules including GBLs, antibiotics and their intermediates. This review is accompanied by four original research articles, which address specific signaling cascades in streptomycetes: Vicente et al., report on the regulatory role of the GBL-receptor-type regulator AlpZ in the kinamycin producer *Streptomyces ambofaciens*. Here, the authors present a molecular dynamics approach, which was applied to investigate the DNA-binding properties of AlpZ to its target DNA sequence and the impact of the potential signaling molecule on receptor-DNA interaction. Misaki et al., explore the repressive function of the pseudo-GBL receptor SrrB on lankacidin and lankamycin production in *Streptomyces rochei* and its hierarchical positioning within the regulatory signaling cascade. The study of Kang et al., shows the involvement of the pleiotropic AdpA-like regulator AdpA_lin_ from *Streptomyces lincolnensis* in lincomycin production and morphological differentiation; whereas Vior et al., report on a so far less studied cluster-situated gene *btmL*, which encodes a protein that acts as a modulator of the biosynthesis of the ribosomally synthesized and post-translationally modified peptide (RiPP) antibiotic bottromycin from *Streptomyces scabies*. Bottromycin shows an activity against clinical relevant pathogens and exhibits a unique chemical structure and mode of action, which makes it a promising drug lead compound. However, production yields in the known producers are very low, which hampered further drug development so far (Vior et al.). Indeed, this is a well-known issue for several drug development approaches, where further advancement is hindered by low production yields. Understanding regulatory principles is a key aspect, which allows the targeted improvement of antibiotic production yields. This aspect is largely covered by two reviews from Xia et al. and Li et al.: The article from Xia et al., summarizes the latest knowledge on regulatory cascades in streptomycetes and their importance for yield improvement applications and metabolite mining purposes, whereas the article from Li et al. concentrates on recent advances of synthetic biology approaches involving also regulatory components for yield improvement strategies. The importance of regulation for antibiotic yield improvement is also highlighted by two original research articles (Park et al.; Yushchuk et al.): Park et al. report on the overexpression of a set of pathway-specific regulatory genes (*nppRI–nppRVI*) in *Pseudonocardia autotrophica*, which significantly improved production of the polyene-like substance NPP B2, whereas the study of Yushchuk et al. extended the genetic toolkit for members of the *Nonomurea* genus, involving the usage of suitable promoters and cluster-situated regulators to improve production yields of the glycopeptide antibiotic A40926 in *Nonomurea gerenzanensis*. As outlined in the review of Xia et al. understanding regulatory signaling can also help to activate silent gene clusters for novel natural compounds discovery. This is underpinned by an original research article from Krause et al. who report on the activating function of the SARP-type regulator PapR2 from *Streptomyces pristinaespiralis* and describe how these type of regulators can be used as general devices to activate antibiotic gene clusters in actinomycetes. Besides several studies that deal with transcriptional regulators, also post-transcriptional regulation is the topic in two research articles: The study of Engel et al. disclosed the post-transcriptional regulatory effect of a small RNA *scr5239* on the primary metabolic enzyme phosphoenolpyruvate carboxykinase PEPK and report on the interconnection between central carbon metabolism and secondary metabolism via this small RNA. Furthermore, the study of Vassallo et al. shows that the conserved small ORF *trpM*, of which the product modulates l-tryptophan biosynthesis, has a regulatory effect on morphological development and antibiotic production in *Streptomyces coelicolor*. TrpM is suggested to interact with the putative cytosol aminopeptidase PepA (SCO2179) via a post-transcriptional and/or post-translational regulatory mechanism, whereby PepA plays a key role in antibiotic production and sporulation. Overall, this collection of articles gives a profound overview on the importance of knowledge on regulation of antibiotic biosynthesis for antibiotic research. We are delighted to present this Research Topic in *Frontiers in Microbiology* and hope that it will promote further research in this area.

## Author Contributions

YM, YL, and ES edited the Frontiers Research Topic on *Regulation of Antibiotic Production in Actinomycetes* and wrote the manuscript. All authors contributed to the article and approved the submitted version.

## Conflict of Interest

The authors declare that the research was conducted in the absence of any commercial or financial relationships that could be construed as a potential conflict of interest.
